# Impact of insurance status on overall survival after cytoreductive surgery and hyperthermic intraperitoneal chemotherapy (CRS-HIPEC)

**DOI:** 10.1515/pp-2020-0105

**Published:** 2020-08-04

**Authors:** Ravi J. Chokshi, Jin K. Kim, Jimmy Patel, Joseph B. Oliver, Omar Mahmoud

**Affiliations:** Division of Surgical Oncology, Rutgers New Jersey Medical School, Newark, NJ, USA; Department of Surgery, Rutgers New Jersey Medical School, Newark, NJ, USA; Department of Radiation Oncology, Rutgers New Jersey Medical School, Newark, NJ, USA

**Keywords:** disparities, hyperthermic intraperitoneal chemotherapy, insurance, outcomes, peritoneal metastasis

## Abstract

**Objectives:**

The impact of insurance status on oncological outcome in patients undergoing cytoreduction and hyperthermic intraperitoneal chemotherapy (CRS-HIPEC) is poorly understood.

**Methods:**

Retrospective study on 31 patients having undergone 36 CRS-HIPEC at a single institution (safety-net hospital) between 2012 and 2018. Patients were categorized as insured or underinsured. Demographics and perioperative events were compared. Primary outcome was overall survival (OS).

**Results:**

A total of 20 patients were underinsured and 11 were insured. There were less gynecologic malignancies in the underinsured (p=0.02). On univariate analysis, factors linked to poor survival included gastrointestinal (p=0.01) and gynecologic malignancies (p=0.046), treatment with neoadjuvant chemotherapy (p=0.03), CC1 (p=0.02), abdominal wall resection (p=0.01) and Clavien–Dindo 3-4 (p=0.01). Treatment with neoadjuvant chemotherapy and abdominal wall resections, but not insurance status, were independently associated with OS (p=0.01, p=0.02 respectively). However, at the end of follow-up, six patients were alive in the insured group vs. zero in the underinsured group.

**Conclusions:**

In this small, exploratory study, there was no statistical difference in OS between insured and underinsured patients after CRS-HIPEC. However, long-term survivors were observed only in the insured group.

## Introduction

Peritoneal metastasis (PM) is a late presentation of malignancies that is characterized by the spread of disease throughout the peritoneum. The cancer subtypes typically seen in this kind of spread are appendiceal, mesothelioma, primary peritoneal, gastrointestinal, and gynecologic malignancies. Amongst diseases with this manner of spread, the survival has shown to be poor without treatment [1]. The main challenge with these types of diseases is the fact that systemic chemotherapy, administered via intravenous route, has poor absorption and activity on the peritoneum. However, with that being the primary option, this has historically been the mainstay of treatment until the emergence of cytoreduction and hyperthermic intraperitoneal chemotherapy (CRS-HIPEC).

Operations for PM are available in highly specialized centers that have the surgical expertise to manage these complex patients. Patients with the means to identify these centers and seek out their professionals have been the recipients of this type of surgery. Hospital and patient costs, morbidity, mortality, and geography have limited access to this operation for many patients [2]. With increasing data supporting CRS-HIPEC and many more centers providing training in the US for these procedures, this has become more widely available. However, access for the underinsured is not clear.

Disparities in cancer diagnosis, treatment, and survival have been demonstrated extensively in the literature [3]. A lack of insurance or poor insurance has shown to lead to delayed diagnoses, fewer treatment options, and decreased survival. The policy of healthcare expansion has been considered a possible remedy for this problem [4]. Unfortunately, long term follow-up and data to support these claims does not yet exist. Due to this lack of information, we investigated outcomes from a safety-net hospital which cares for both insured and underinsured patients.

To our knowledge this is the first study comparing outcomes from CRS-HIPEC between patients with private insurance and underinsured patients (Medicare, Medicaid, Charity Care, and Self-Pay). We investigated the outcomes from this operation in a cohort from the largest provider of uncompensated care in NJ to see if disparities exist.

## Materials and methods

Under the Rutgers Institutional Review Board (IRB) approved protocol, we performed a retrospective analysis on patients who underwent CRS-HIPEC for treatment of various peritoneal malignancies between January 2012 and September 2018 at University Hospital in Newark, NJ. This institution is the largest provider of uncompensated care in New Jersey that functions as a safety-net hospital.

We performed a case-control study on the patients’ risk factors and performed a cohort study to examine their outcomes. The patients were categorized into insurance statuses; underinsured and insured. Underinsured consisted of patients with Medicare, Medicaid, charity care, or self-pay. Insured patients were categorized as patients with private insurance. Demographics, tumor subtypes, operative details, comorbidities, length of stay (LOS), extent of disease, resectability, complications, morbidity, mortality, and survival data were abstracted from the medical record. Demographics included age, gender, and race. Tumor subtypes were categorized into primary peritoneal, gastrointestinal, and gynecological origins. The primary peritoneal category was composed of tumors that most frequently were found to have peritoneal metastases. Operative details that were reviewed consisted of estimated blood loss (EBL) and operative time (OT). Comorbidities that were reported on were presence of diabetes, hypertension, or smoking. Outcomes related to CRS-HIPEC surgery have been correlated to the amount of disease present and the success of resection. These are characterized by use of Peritoneal Carcinomatosis Index (PCI) and Complete Cytoreduction score (CC). Complications were classified as early (<30 days) or late (>30 days). Morbidities were calculated using the Clavien–Dindo score, which classifies complications from a scale of 1 (minor requiring no intervention) to 5 (mortality), and we stratified this into minor [1], [5] or major [6], [7] morbidity. Survival data was calculated using an end point of 09/18/18 as still alive. The groups were compared using t -test, Fisher’s exact test, and χ^2^-testing where appropriate. Utilizing R statistical package, survival data was analyzed using Kaplan-Meier curves as well as Univariate and Multivariate Cox Proportional Hazards models.

## Results

A total of 36 CRS-HIPEC procedures were done between 2012 and 2018 on 31 unique patients. Two patients had undergone multiple CRS-HIPEC. For cases of more than one HIPEC, the data from those patients was taken from the index case. Of this group, 11 patients were insured and 20 patients were underinsured. Table 1 shows the preoperative, perioperative, and postoperative data for both groups. There were no differences in age, gender, or race between the two groups. There was also no significant difference between any of the medical comorbidities between the groups.

**Table 1:** Baseline characteristics stratified by insurance.

	Underinsured (n=20)	Insured (n=11)	p-Value	OR	95% Lower CI	95% Upper CI
Age, years	57 (36–76)	60 (46–74)	0.43			
Female gender	15 (75%)	10 (91%)	0.28	0.30	0.03	2.97
Race						
White vs Non-white	7 (35%)	7 (64%)	0.13	0.31	0.07	1.43
Pathology						
Primary (Appendix, Primary peritoneal, Mesothelioma)	10 (50%)	2 (18%)	0.08	4.5	0.77	26.29
GI	8 (40%)	4 (36%)	0.84	1.17	0.26	5.33
GYN	2 (10%)	5 (45%)	0.02	0.13	0.02	0.88
Signet ring	1 (5%)	4 (36%)	0.02	0.09	0.01	0.97
EBL, mL	801 (10–2500)	814 (50–3000)	0.96			
OR time, minutes	548 (183–828)	497 (131–840)	0.49			
DM	3 (15%)	4 (36%)	0.17	0.31	0.05	1.75
HTN	7 (35%)	4 (36%)	0.94	0.94	0.20	4.37
Smoker	10 (50%)	5 (45%)	0.26	1.2	0.27	5.25
PCI	18.2 (2–33)	16.6 (2–32)	0.67			
CC						
0	16 (80%)	9 (82%)	0.90	0.89	0.14	5.85
1	3 (15%)	2 (18%)	0.82	0.79	0.11	5.66
2	1 (5%)	0 (0%)	1	1.77	0.07	47.14
Mean ICU length of stay (days)	24 (0–60)	9 (0–35)	0.41			
Mean hospital length of stay (days)	18.5 (4–78)	18.7 (6–41)	0.96			
Early complication	13 (65%)	5 (45%)	0.29	2.23	0.50	10
Late complication	8 (40%)	2 (18%)	0.21	3	0.51	17.69
Morbidity – Clavien–Dindo	16 (80%)	8 (64%)	0.64	1.50	0.27	8.38
I-II	6	2	0.47	1.93	0.32	11.74
III-IV	9	5	0.98	0.98	0.22	4.3
Mortality 30 day	0 (0%)	0 (0%)	1	0.56	0.01	30.2
Mortality 90 day	1 (5%)	1 (9%)	1	0.53	0.03	9.33
Survival days (Mean)	1173 (97–2335)	978 (103–1642)	0.38			

EBL, estimated blood loss.

The pathologic subtypes were different between both groups; there were less gynecologic malignancies in the underinsured group (OR 0.13, CI .02–0.88, p=0.02), while there trended to more primary peritoneal malignancies in the underinsured group compared to the privately insured (OR 4.5, CI 0.77–26.29, p=0.08). Signet ring pathology, which has been postulated to lead to poorer outcomes, was lower in the underinsured group (OR 0.09, CI 0.01–0.97, p=0.02). The groups demonstrated similar PCI scores (18.2 vs. 16.6; p=0.067) and CC 0 (OR 0.89, CI 0.14–5.85), 1 (OR 0.79, CI 0.11–5.66), and 2 (OR 1.77, CI 0.07–47.14) demonstrated no statistical significance, suggesting no differences in the peritoneal disease burden and completeness of cytoreductive surgery.

**Figure 1:**
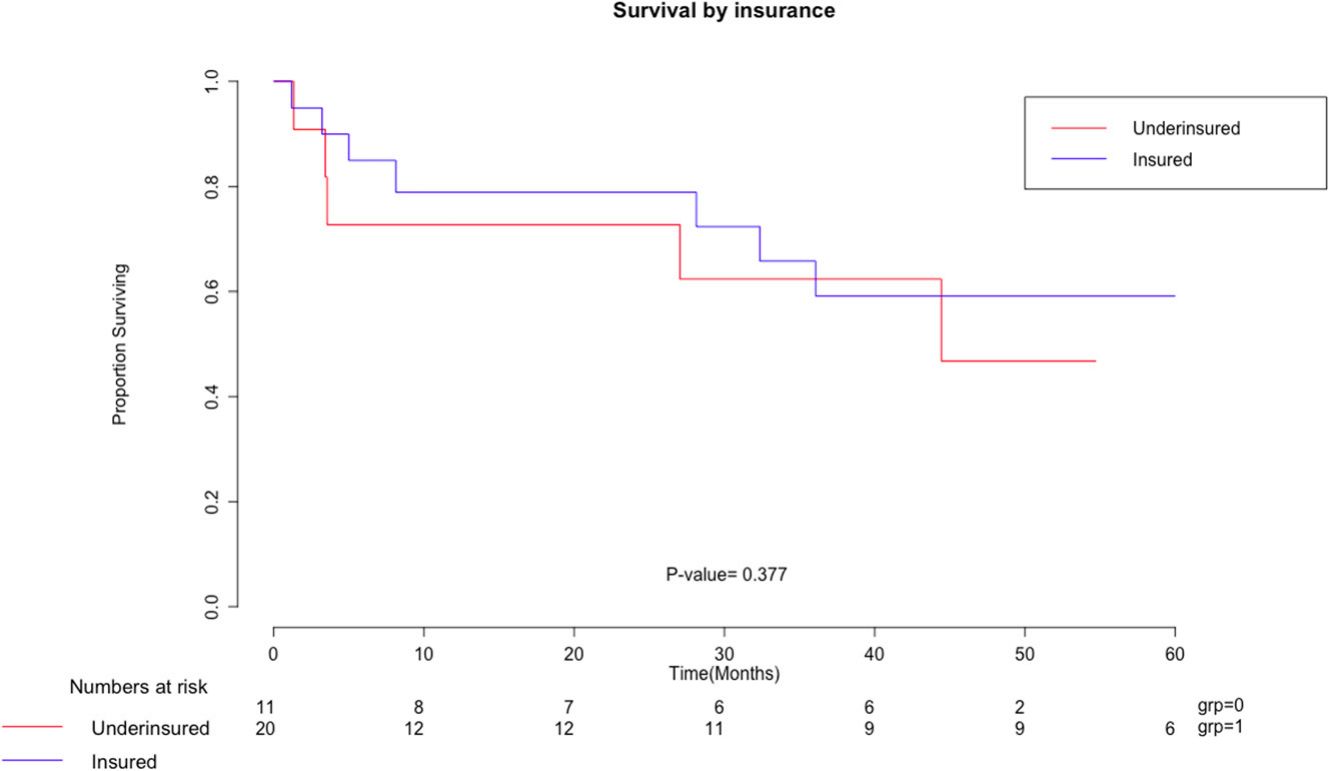
Kaplan Meier survival curve by insurance status.

Perioperative outcomes were similar between both groups. No significant differences were found between the underinsured and insured groups for OT (584 vs. 497 min) or EBL (801 vs. 814 mL). LOS in the hospital (18.5 vs. 18.7 days) and mean intensive care unit (ICU) LOS, for those who required it, (24 vs. 9 days) were not statistically significant. We wondered whether there would be differences in postoperative morbidity and mortality between the two groups. Early complications were considered in the first 90 days and late complications after 90 days from the index operation. We did not observe statistical differences in early or late complications. Nor were any differences seen in minor or major morbidity by Clavien–Dindo classification. We did not observe differences in 30 day or 90 day postop mortality in the two groups. Survival in the underinsured was a mean of 1,173 days and in the insured was 978 days (p=0.38). Figure 1 is the Kaplan–Meier curve comparing overall survival (OS) for the insured and underinsured, demonstrating no survival difference between the two groups.

Table 2 demonstrates the Cox proportional hazard ratio of factors associated with OS. Insured individuals had no benefit in survival in either univariate (HR 0.74, p=0.61) or multivariate analysis (HR 0.97, p=0.98) compared to underinsured individuals. Age and sex were not associated with OS. Comorbidities were added into the model to see if there was any effect on survival. Diabetes, congestive heart failure, hypertension, body mass index, and the presence of liver and renal disease were evaluated. We suspected these factors may lead to a difference in survival in the univariate analysis. However, there were no statistically significant differences. Preoperative factors that had a significant impact on survival in univariate analysis included gastrointestinal pathology (HR 18.42, p=0.01) and gynecologic pathology (HR 9.41, p=0.046), compared to primary peritoneal malignancy, and treatment with neoadjuvant chemotherapy (HR 3.62, p=0.03). Neoadjuvant chemotherapy was defined as preoperative chemotherapy administered with the intent to shrink tumor or test tumor biology in order to proceed with CRS-HIPEC. Adjuvant chemotherapy was defined as the addition of chemotherapy after CRS-HIPEC. We defined definitive chemotherapy as a group that had completed full treatment of systemic therapy and then was referred for CRS-HIPEC. Only treatment with neoadjuvant chemotherapy remained significant on multivariate analysis with an association with worse survival (HR 37.5, p=0.01).

**Table 2.** Prognostic factors for survival on univariate and multivariate analysis for the entire cohort.

Variable	Univariate		Multivariate	
	HR	p-Value	HR	p-Value
Demographics				
Age	0.98	0.53		
Sex	1.43	0.66		
Insurance Group	0.74	0.61		
Race	0.91	0.82		
Pathology				
Primary (ref)	1	–	1	–
Gastrointestinal	18.42	0.01	1.64E+10	1
Gynecologic	9.41	0.046	4.65E+08	1
Signet ring	2.75	0.14		
Perioperative factors				
Hospital Length of stay	1.03	0.29		
ICU length of stay	1	0.89		
Received definitive chemo	1.74	0.35		
Received neoadjuvant chemo	3.62	0.03	3.75E+01	0.01
Received adjuvant chemo	1.59	0.46		
Preop albumin	1.05	0.90		
Preop hemoglobin	0.85	0.25		
CC				
0 (ref)	1	–	1	–
1	4.22	0.02	1.68E+00	0.68
2	4.03	0.20	4.55E+10	1
Comorbidities				
Diabetes	0.95	0.94		
Smoking	1.16	0.69		
Congestive heart failure	1.28E-08	1		
Hypertension	0.43	0.20		
Body mass index	0.94	0.22		
Renal disease	4.20E-09	1		
Liver disease	1.40E-08	1		
Resected organs				
Splenectomy	0.99	0.99		
Gastrectomy	1.26E-08	1		
Small bowel resection	1.98	0.31		
Colectomy	1.09	0.88		
Pancreatectomy	2.05	0.24		
Cholecystectomy	5.48	0.12		
Hysterectomy	1.13	0.84		
Omentectomy	0.95	0.94		
Peritonectomy	0.92	0.90		
Abdominal wall resection	5.75	0.01	3.30	0.02
Ostomy	2.22	0.17		
Complications				
Clavien–Dindo 3-4	1.93	0.01	2.25	0.15
Early Complication	3.79E+08	1		
Late Complication	0.60	0.45		

We hypothesized that the more extensive resections with higher morbidity would affect survival. However, univariate analysis of the type of resections (splenectomy, gastrectomy, small bowel resection, colectomy, pancreatectomy, cholecystectomy, hysterectomy, omentectomy, abdominal wall resection, and presence of ostomy) was not associated with OS. Perioperative features that had a significant impact on survival in univariate analysis included CC1 (HR 4.22, p=0.02) and abdominal wall resection (HR 5.75, p=0.01). Multivariate regression analysis on abdominal wall resections demonstrated an HR of 3.3 (p=0.02) demonstrating its negative effect on survival in this model, while CC1 was no longer significant. Postoperative measures demonstrated Clavien–Dindo type 3-4 complications were significant in univariate regression model with HR of 1.93 (p=0.01), but no longer when placed into a multivariate regression model (HR 2.25, p=0.15).

## Discussion

The focus on access and disparities in cancer care has revolved around the presence or absence of medical insurance coverage. This study used the terms insured and underinsured to describe these patients. Underinsured consisted of patients who were classified as self-pay, charity-care, Medicaid, and Medicare. This effectively allowed separation of the two groups into those who can pay for care as opposed to those who cannot.

Disparities amongst patients who are uninsured or underinsured have been highlighted in many oncologic studies [8]. However, to date, there has been no examination of this related to CRS-HIPEC for patients with PM. Historically, these operations were confined to specialized centers and much of its work was done on protocol. With increased data supporting its use, it has become much more widely available. Expansion of its use has been demonstrated by the large number of clinical trials that are currently ongoing [9].

Despite its expansion of use as a technique to address PM patients, most centers are not able to offer this time consuming and complex surgery. The presence of protocols, trained personnel, resources, and financial costs act as barriers to establishing a program. While literature highlights socioeconomic disparity in access to cancer care, we found no significant differences between privately and underinsured patients with colorectal malignancies at this institution [10]. However, since PM is a much less commonly encountered problem and requires expertise, we sought out to see if differences in outcomes exist due to insurance status.

CRS-HIPEC patients’ insurance status at other institutions is not known. Based on published studies, we assumed most of these patients held private insurances. At our institution, we treated a mix of underinsured and insured patients. Due to the small numbers in the study, we did not see any difference between the insured and underinsured groups. In addition, demographic data including age, gender, and sex were similar between both groups. We did note that amongst the pathologic diagnoses, underinsured patients trended toward having a larger number of primary peritoneal malignancy in comparison to the insured group. The insured group had significantly more gynecologic malignancies than the underinsured group. These differences may be explained by the practice and referral patterns in the community. In univariate model, gastrointestinal pathology demonstrated the poorest survival. This is expected due to the varying malignancy subtypes within this group (gastric, small intestine, and colorectal) [11]. Each of these subtypes has been shown to have poorer survival in comparison to primary pathologic types and gynecologic malignancies. The pathology subtype differences were not borne out in the multivariate model when looking at survival.

Signet ring pathology has long been associated with poorer outcomes and survival [12]. The statistically significant difference of more signet ring pathology in the insured group may arise from the fact that these are typically more aggressive and were referred to the tertiary care center for evaluation. Statistically significant survival differences were not found in the univariate model likely because of the small sample size.

CRS-HIPEC operations are complicated and time intensive. They require careful evaluation of the abdomen with resection of any visible disease including involved organs and the peritoneum. PCI and CC have been shown to directly affect survival [13]. Based on the PCI and ability to completely cytoreduce, the extent of these operations can vary. Comparing the two groups, there were no differences in PCI, CC, OTs, or EBL. Univariate analysis did demonstrate survival differences between CC. This was expected since CRS-HIPEC survival has been shown to be the most beneficial when CC0 can be achieved [13]. Post-operatively, all patients are required to be admitted for close monitoring. Some may require either ICU bed and/or a monitored bed. The ICU LOS and hospital LOS did not differ between the two groups. In the univariate model, there was no statistical significance on survival.

Due to the radical nature of these operations, major morbidity and mortality are not uncommon [14]. The literature varies on the extent, but recent studies have demonstrated major complication rate at about 20% with 30-day mortality at 2.3% [15]. Our study organized complications into an early and late stage. Both groups showed high rates of early and late complications but with no statistical significance. In the univariate analysis, there was no difference in survival related to complications. Morbidity was analyzed using the Clavien–Dindo scale. In our analysis, since we looked at mortality, we excluded Clavien–Dindo 5 in the major morbidity. There was no significant difference in minor or major morbidity when comparing the underinsured and insured groups. Univariate analysis did demonstrate a statistically significant poorer survival in patients with higher Clavien–Dindo scores. This was not seen in the multivariate model. No 30 day mortality existed in this study. There was no statistical difference between the 90 day mortality in the two groups. Survival data was compared between the underinsured and insured groups but we did not see a statistical difference.

The role of chemotherapy as neoadjuvant, adjuvant, or definitive was also added to the model. Each of these groups had very different characteristics. Univariate analysis demonstrated a statistically significant poorer survival in patients who underwent neoadjuvant chemotherapy. This continued into the multivariate model and demonstrated survival significance. Review of the literature shows that there is no clear consensus on this type of therapy. Varying malignancy subtypes have had different outcomes. Many of the studies suggest that this operation is safe but have not commented on survival [16], [17]. This may prove to be an area where further research may glean as to why these findings are present. We hypothesize that the neoadjuvant chemotherapy does not provide enough time to optimally decide if patients will benefit from CRS-HIPEC. In contrast, definitive chemotherapy is given for a longer period which may allow separation of patients who respond and not progressed making their disease more amenable to CRS-HIPEC. Adjuvant chemotherapy provides systemic treatment to patients who have already been optimally cytoreduced and, in these cases, have less disease allowing for better penetration of therapy.

The extent of surgical resection including organs and peritoneum were examined in the model. Of the surgical resections, only abdominal wall resection was associated with poorer survival in the univariate analysis. In the multivariate model, this was also statistically significant. This is consistent with already published data that demonstrates abdominal wall reconstruction at the time of CRS-HIPEC due to radial resections may lead to higher grade complications and delay administration of systemic chemotherapy [18]. The absence of adjuvant therapy in certain malignancies leads to significantly poorer survival.

There are several limitations to this study. These include a small number of patients, a heterogeneous CRS-HIPEC patient group, lack of longterm mortality data, and lack of comparison data from other studies about insurance status of their patients. This study was exploratory in nature and not adequately powered to detect differences in survival between insured and underinsured patients. However, we observed that six patients in the insured group were still alive vs. zero in the underinsured group during follow up in our study, and speculate that a larger study may be able to determine the impact of insurance on OS in CRS-HIPEC patients.

This manuscript adds data not available in the literature about CRS-HIPEC outcomes in underinsured and insured groups. As an urban tertiary care hospital which acts a safety-net hospital, we have not identified any significant disparities in CRS-HIPEC outcomes related to insurance status.
